# Serine phosphorylation of cortactin is required for maximal host cell invasion by *Campylobacter jejuni*

**DOI:** 10.1186/1478-811X-11-82

**Published:** 2013-11-04

**Authors:** Derrick R Samuelson, Michael E Konkel

**Affiliations:** 1 School of Molecular Biosciences, Washington State University, College of Veterinary Medicine, Life Sciences Bldg. Room 302c, Pullman, Washington 99164-7520, USA

**Keywords:** Bacterial pathogenesis, Effector protein, Invasion, Erk 1/2, N-WASP

## Abstract

**Background:**

*Campylobacter jejuni* causes acute disease characterized by severe diarrhea containing blood and leukocytes, fever, and abdominal cramping. Disease caused by *C. jejuni* is dependent on numerous bacterial and host factors. *C. jejuni* invasion of the intestinal epithelial cells is seen in both clinical samples and animal models indicating that host cell invasion is, in part, necessary for disease. *C. jejuni* utilizes a flagellar Type III Secretion System (T3SS) to deliver the *Campylobacter* invasion antigens (Cia) to host cells. The Cia proteins modulate host cell signaling leading to actin cytoskeleton rearrangement necessary for *C. jejuni* host cell invasion, and are required for the development of disease.

**Results:**

This study was based on the hypothesis that the *C. jejuni* CiaD effector protein mediates Erk 1/2 dependent cytoskeleton rearrangement. We showed that CiaD was required for the maximal phosphorylation of Erk 1/2 by performing an immunoblot with a p-Erk 1/2 specific antibody and that Erk 1/2 participates in *C. jejuni* invasion of host cells by performing the gentamicin protection assay in the presence and absence of the PD98059 (a potent inhibitor of Erk 1/2 activation). CiaD was also found to be required for the maximal phosphorylation of cortactin S405 and S418, as judged by immunoblot analysis. The response of human INT 407 epithelial cells to infection with *C. jejuni* was evaluated by confocal microscopy and scanning electron microscopy to determine the extent of membrane ruffling. This analysis revealed that CiaD*,* Erk 1/2, and cortactin participate in *C. jejuni*-induced membrane ruffling. Finally, cortactin and N-WASP were found to be involved in *C. jejuni* invasion of host cells using siRNA to N-WASP, and siRNA to cortactin, coupled with the gentamicin protection assay.

**Conclusion:**

We conclude that CiaD is involved in the activation of Erk 1/2 and that activated Erk 1/2 facilitates *C. jejuni* invasion by phosphorylation of cortactin on serine 405 and 418. This is the first time that cortactin and N-WASP have been shown to be involved in *C. jejuni* invasion of host cells. These data also provide a mechanistic basis for the requirement of Erk 1/2 in *C. jejuni-*mediated cytoskeletal rearrangement.

## Lay abstract

*Campylobacter jejuni* uses the flagellum as a Type III Secretion System (T3SS). A subset of proteins are exported from the flagellum and delivered to the cytosol of host cells where they modify host cell signaling events to promote bacterial invasion. Here we report that *C. jejuni,* which is the leading bacterial cause of food-borne disease worldwide, usurps the host cell signaling proteins Erk 1/2 and cortactin. We show that the *C. jejuni* CiaD protein is required for the invasion of host cells and for the activation of Erk 1/2 (a host cell kinase) and cortactin (a cellular scaffolding protein). The characterization of a virulence protein and the identification of a novel host cell signaling pathway exploited by *C. jejuni* provides a significant advancement in the understanding of *C. jejuni* pathogenesis.

## Background

Cortactin is an actin-binding protein that plays an integral role in the regulation and dynamics of the actin cytoskeleton. Cortactin has emerged as a key cellular protein that microbes readily subvert during the establishment of infection [1]. To date, cortactin has been demonstrated to be essential for the development of disease by numerous bacterial pathogens. While several pathogens, including *Shigella*, *Neisseria*, *Rickettsia*, *Chlamydia*, *Staphylococcus*, *Listeria*, *Helicobacter*, *Escherichia,* and *Coxiella,* require Src-mediated tyrosine phosphorylation of cortactin for host cell invasion, the mechanism of cortactin activation has only been partially elucidated or is, in most instances, not known [1-8]. The role of various actin cytoskeleton regulators, including Erk 1/2 and cortactin, in *C. jejuni* invasion has not been elucidated.

*Campylobacter jejuni* is a Gram-negative bacterial pathogen that causes acute disease characterized by severe diarrhea. *C. jejuni* causes ~1.4 to 2.3 million cases of gastroenteritis in the United States each year [9]. Guillain–Barré syndrome (GBS), an autoimmune disease affecting the peripheral nervous system, can be a possible sequelae associated with certain strains of *C. jejuni*[10]. Motility, adherence, invasion, intracellular survival, and toxin production have all been shown to contribute to the severity of *C. jejuni*-mediated disease, illustrating the fact that disease is a multifactorial process [11-16].

Maximal cell invasion requires the *Campylobacter* invasion antigens (Cia) [14]. The Cia proteins are exported from the bacterium’s flagellar Type III Secretion System (T3SS) and are delivered to the target host cell, where they presumably modify host cell regulatory proteins to promote *C. jejuni*-host cell entry and intracellular survival. Co-culture of *C. jejuni* with either host cells or host-like conditions results in increased expression of the genes encoding the Cia proteins [17,18]. To date, four Cia proteins have been identified, designated CiaB, CiaC, CiaD, and CiaI [11,16,19,20]. The importance of the Cia proteins in campylobacteriosis has been demonstrated using *in vivo* studies with a *ciaB* mutant, which is deficient in the secretion of all of the Cia proteins [15]. Piglets inoculated with a *C. jejuni* wild-type strain develop severe diarrhea within 24 hours of infection and exhibited major histological abnormalities, such as villus blunting and production of exudates in the lumen. In contrast, piglets inoculated with the *ciaB* mutant did not developed diarrhea until 3 days post infection and only exhibited minor histological lesions. Piglets inoculated with a *ciaB* mutant harboring a wild-type copy of the *ciaB* gene exhibited clinical signs of disease similar to piglets inoculated with the *C. jejuni* wild-type strain [21]. Given that the Cia proteins contribute to the development of *C. jejuni*-mediated enteritis, additional work is warranted to further dissect the functions of these proteins.

We recently identified a secreted protein, which we termed CiaD (Cj0788), that is exported from the flagellum and delivered to the cytosol of host cells. We found that CiaD activates the host cell kinases p38 and Erk 1/2, resulting in the secretion of interleukin-8 (IL-8) from host cells. Similarly, we found that CiaD-mediated activation of p38 and Erk 1/2 are required for maximal invasion of host cells by *C. jejuni.* CiaD function within host cells is dependent on a mitogen-activated protein (MAP) kinase-docking motif. Finally, CiaD was found to contribute to the development of disease, as evidenced by gross pathology and histopathology of tissues from IL-10 knockout mice inoculated with a *C. jejuni* wild-type strain, *ciaD* mutant, and *ciaD* complemented isolate [22]. While it is known that CiaD contributes to *C. jejuni* invasion of host cells, the details of the molecular mechanisms of *C. jejuni-*host cell invasion are incomplete.

The goal of this study was to determine the role of CiaD in *C. jejuni* host cell invasion. We hypothesized that the *C. jejuni* effector protein CiaD contributes to bacterial invasion by stimulation of Erk 1/2 and the phosphorylation of cortactin. We sought to identify the role of Erk 1/2 and cortactin in *C. jejuni* invasion of host cells. More specifically, we sought to determine if the phosphorylation of cortactin is necessary for *C. jejuni* invasion of host cells, and whether CiaD contributes to the phosphorylation of cortactin.

## Results

### Erk 1/2 and the *C. jejuni* CiaD effector protein are required for maximal invasion of human INT 407 epithelial cells

**Figure 1:**
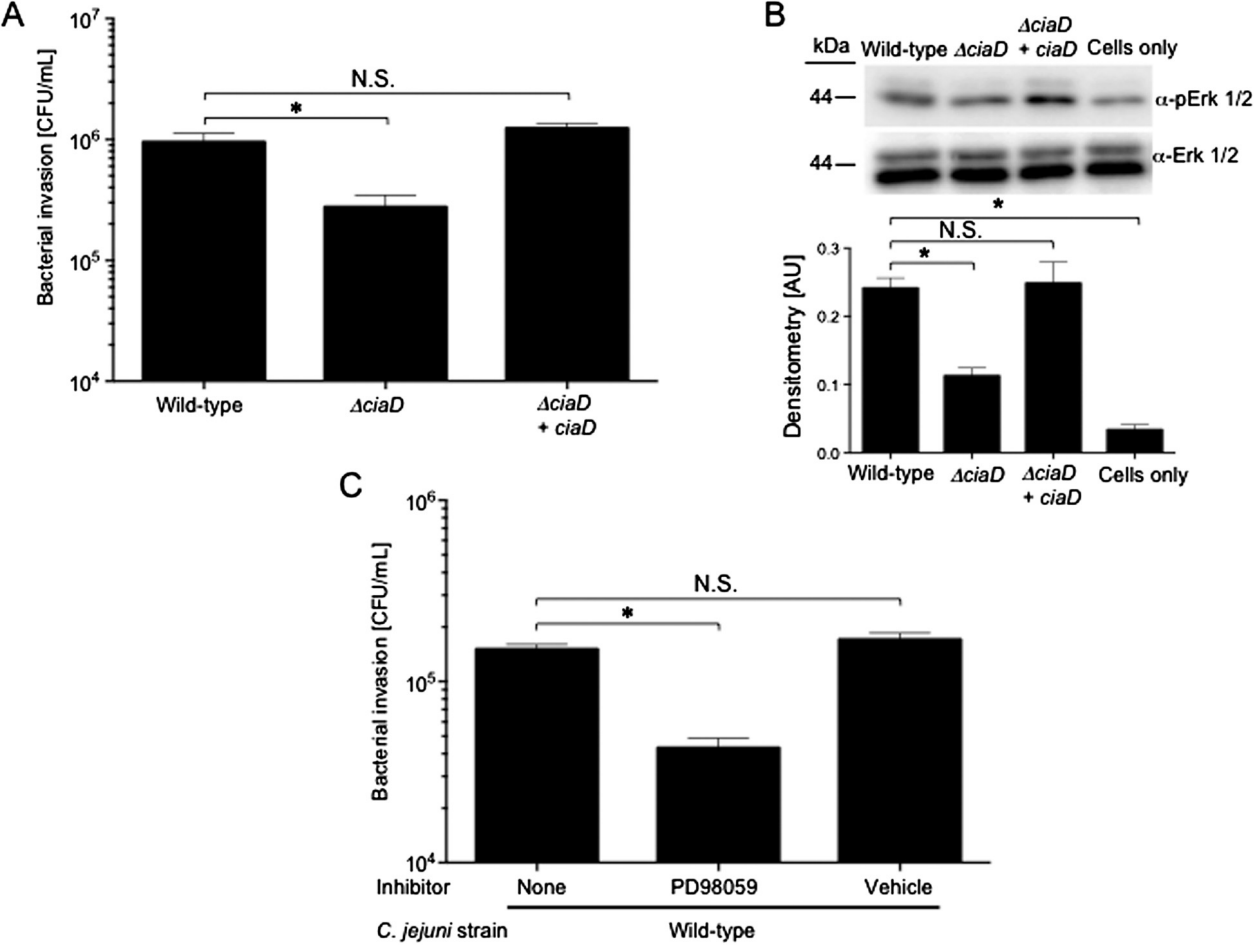
**CiaD and Erk 1/2 are required for maximal *C. jejuni *invasion. A**. The *C. jejuni ciaD* mutant is deficient in maximal invasion of INT 407 cells. Invasion of INT 407 cells was assessed using a gentamicin protection assay as outlined in Methods. **B**. Maximal activation of Erk 1/2 requires CiaD*.* INT 407 cells were infected with the various isolates of *C. jejuni* for 45 min and cell lysates were evaluated via immunoblot. Blots were probed with phospho-specific antibodies to Erk 1/2. All blots were stripped and re-probed with an α-Erk 1/2 antibody. Molecular mass size standards, in kilodaltons (kDa), are indicated on the left. Densitometry of pErk 1/2 is shown as the ratio of p-Erk 1/2 to t-Erk 1/2 for each sample. **C**. Erk 1/2 kinase activation is required for maximal bacterial invasion. PD98059, an inhibitor of Erk 1/2 activation, was added to INT 407 cells for 30 min prior to inoculation with a *C. jejuni* wild-type strain, and bacterial invasion was assessed. The asterisk indicates a significant reduction compared to the value obtained for the *C. jejuni* wild-type strain, as judged by one-way ANOVA followed by post-hoc Dunnett’s analysis (*P* < 0.01). N.S. indicates no significant difference. Results are shown as means ± SEM.

Experiments were initially performed to determine if CiaD contributes to the activation of the Erk 1/2 signaling pathway. Consistent with previous work [22], we found that CiaD is required for maximal invasion of host INT 407 cells (Figure 1A) and contributes to the complete activation of the host cell kinase Erk 1/2 (Figure 1B). INT 407 cells are a human epithelial cell line [23]. To determine if Erk 1/2 is involved in bacterial invasion, we performed a gentamicin protection assay in the presence of the MEK 1/2 inhibitor PD98059 (a potent inhibitor of Erk 1/2 activation). Inhibition of Erk 1/2 activation was found to significantly decrease the number of *C. jejuni* internalized (Figure 1C), which is consistent with previous reports [24,25]. These results demonstrate that CiaD and Erk 1/2 are necessary for maximal host cell invasion by *C. jejuni*.

### CiaD is required for host cell membrane ruffling independent of Rho GTPase activation

**Figure 2:**
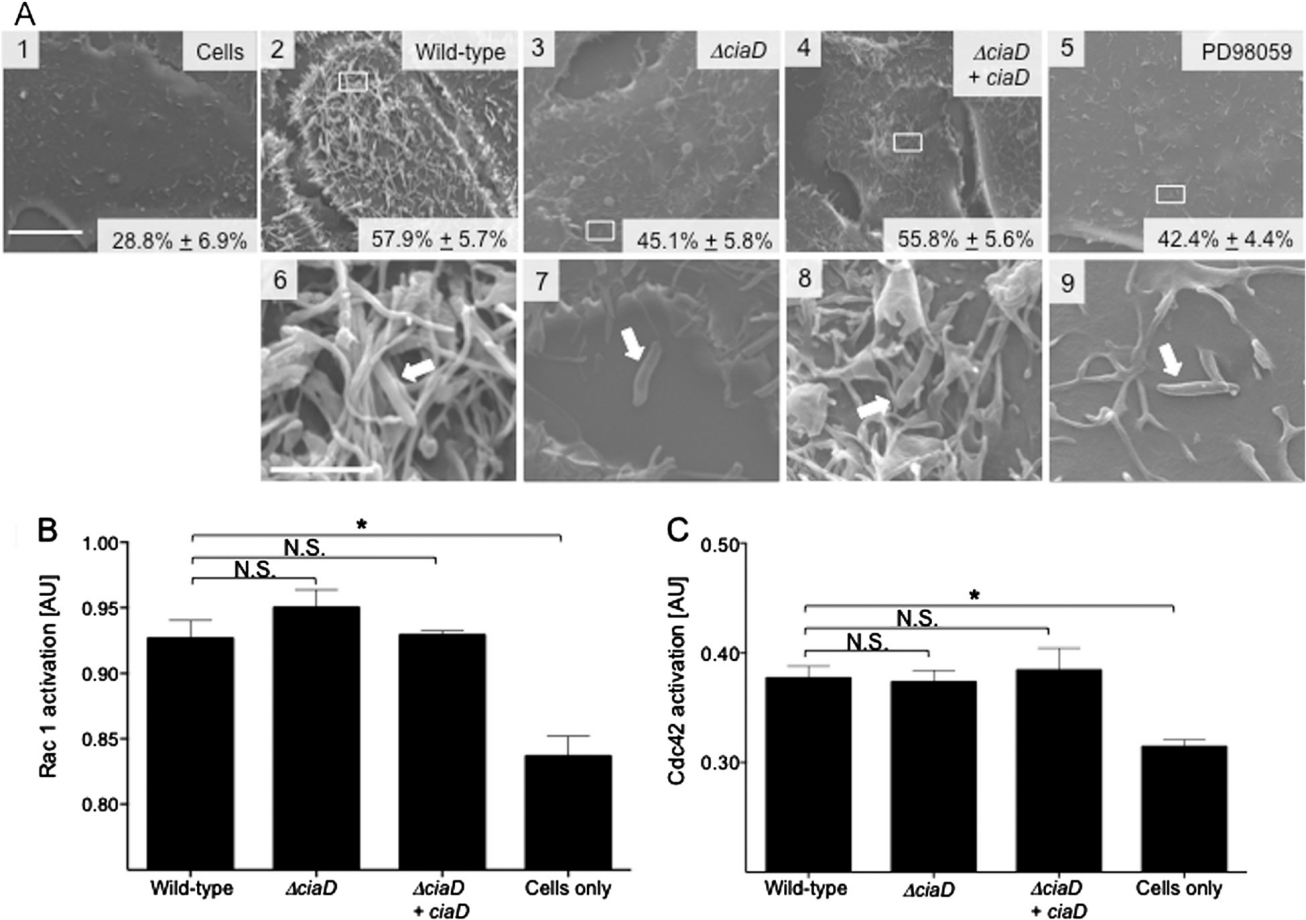
**CiaD induced host cell membrane ruffling is Rho GTPase independent. A**. Representative scanning electron microscopy images of INT 407 cells; uninfected (Panel 1), infected with a *C. jejuni* wild-type strain (2 and 6), *ciaD* mutant (3 and 7), *ciaD* complemented isolate (4 and 8), and cells infected with a *C. jejuni* wild-type strain that had been pretreated with PD98059, an inhibitor of Erk 1/2 activation (5 and 9). Arrows in higher magnification images show bacteria in direct contact with host cells (6, 7, 8, and 9). INT 407 cells infected with the *C. jejuni* wild-type strain or the *ciaD* complemented isolate show extensive membrane ruffling (6 and 8), and INT 407 cells infected with the *ciaD* mutant or cells infected with a *C. jejuni* wild-type strain that had been pretreated with PD98059 display little host cell membrane ruffling (7 and 9). Images are shown at a magnification of 7,000× with a 10 μM scale bar (1–5), and 50,000× with a 2 μM scale bar (6–9). Boxes indicate the area of the INT 407 cell that is shown in the 50,000× panel. Indicated within each panel is the percent of host cell that display membrane ruffling*.***B**. Rac1 activation in host cells infected with *C. jejuni*. Whole cell lysates were processed after 15 min of infection and analyzed for activated Rac1 by G-LISA™. The mean ± SEM of total active Rac1 is indicated in relative optical density. **C**. Cdc42 activation in host cells infected with the various *C. jejuni* strains was assessed by G-LISA™. The mean ± SEM of total active Cdc42 is indicated in relative optical density. The asterisks indicate a significant difference (*P* < 0.01) in the level of Rac1 or Cdc42 activation in cells infected with the *C. jejuni* wild-type strain, as judged by one-way ANOVA followed by post-hoc Dunnett’s analysis. N.S. indicates no significant difference.

Three Rho GTPases (*i.e.,* Rho, Rac1, and Cdc42) are involved in the regulation and dynamic rearrangement of the actin cytoskeleton [26]. *C. jejuni* invasion of host cells is accompanied by the activation of the Rho GTPases Rac1 and Cdc42 [27,28]. Rac1 is involved in the formation of lamellipodia and Cdc42 is involved in the formation of filopodia [26]. We will refer to lamellipodia and filopodia extensions, which are membrane protrusions associated with reorganization of actin microfilaments, as membrane ruffles throughout the manuscript. Experiments were performed to assess membrane ruffling of INT 407 cells upon infection with the *C. jejuni* wild-type strain, *ciaD* mutant, and *ciaD* mutant expressing a wild-type copy of *ciaD in trans* (*ciaD* complemented isolate), as well as cells infected with a *C. jejuni* wild-type strain that had been pretreated with the MEK 1/2 inhibitor PD98059 that blocks Erk 1/2 activation. Representative scanning electron microscopy (SEM) images of the various *C. jejuni* strains interacting with host INT 407 cells are shown in Figure 2A1-9. Both flagellated and non-flagellated bacteria were visualized bound to the host cells. The observation of non-flagellated bacteria bound to the cells was presumably due to the method of fixation, as all of the bacterial isolates were highly motile as judged by motility assays (not shown). We observed that 28.8% ± 6.9% of untreated and uninfected INT 407 cells had membrane ruffling (Figure 2A1). In contrast, 57.9% ± 5.7% of INT 407 cells had pronounced membrane ruffling when inoculated with a *C. jejuni* wild-type strain (Figure 2A2). Inoculation of INT 407 cells with the *ciaD* mutant resulted in membrane ruffling in 45.1% ± 5.8% of the cells (Figure 2A3), whereas inoculation of cells with the *ciaD* complemented isolate resulted in membrane ruffling in 55.8% ± 5.6% of the cells (Figure 2A4). Pretreatment of host cells with the Erk 1/2 inhibitor reduced the percentage of host cells with membrane ruffling to 42.4% ± 4.4% (Figure 2A5). Based on these data, we concluded that maximal membrane ruffling of host INT 407 cells requires CiaD and Erk 1/2.

Given that the *C. jejuni ciaD* mutant was found to be deficient in stimulating membrane ruffling, we investigated whether there was a defect in Rho GTPase activation. The *ciaD* mutant exhibited normal Rac1 (Figure 2B) and Cdc42 (Figure 2C) activity when compared to the *C. jejuni* wild-type strain, as determined by G-LISA**™***.* The fact that the activation levels of the Rho GTPases are not changing in the *C. jejuni ciaD* mutant was interesting, as there were clear reductions in bacterial invasion and host cell membrane ruffling. These data indicate that activated Rac1 and Cdc42 require assembly and/or activation of scaffold or accessory proteins to facilitate lamellipodia and filopodia extensions. Given the complexity of the *C. jejuni-*mediated invasion complex, we chose to focus on the role of CiaD-mediated Erk 1/2 activation and the potential targets of Erk 1/2 that participate in membrane ruffling.

### CiaD mediated Erk 1/2 activation is required for cortactin serine phosphorylation

**Figure 3:**
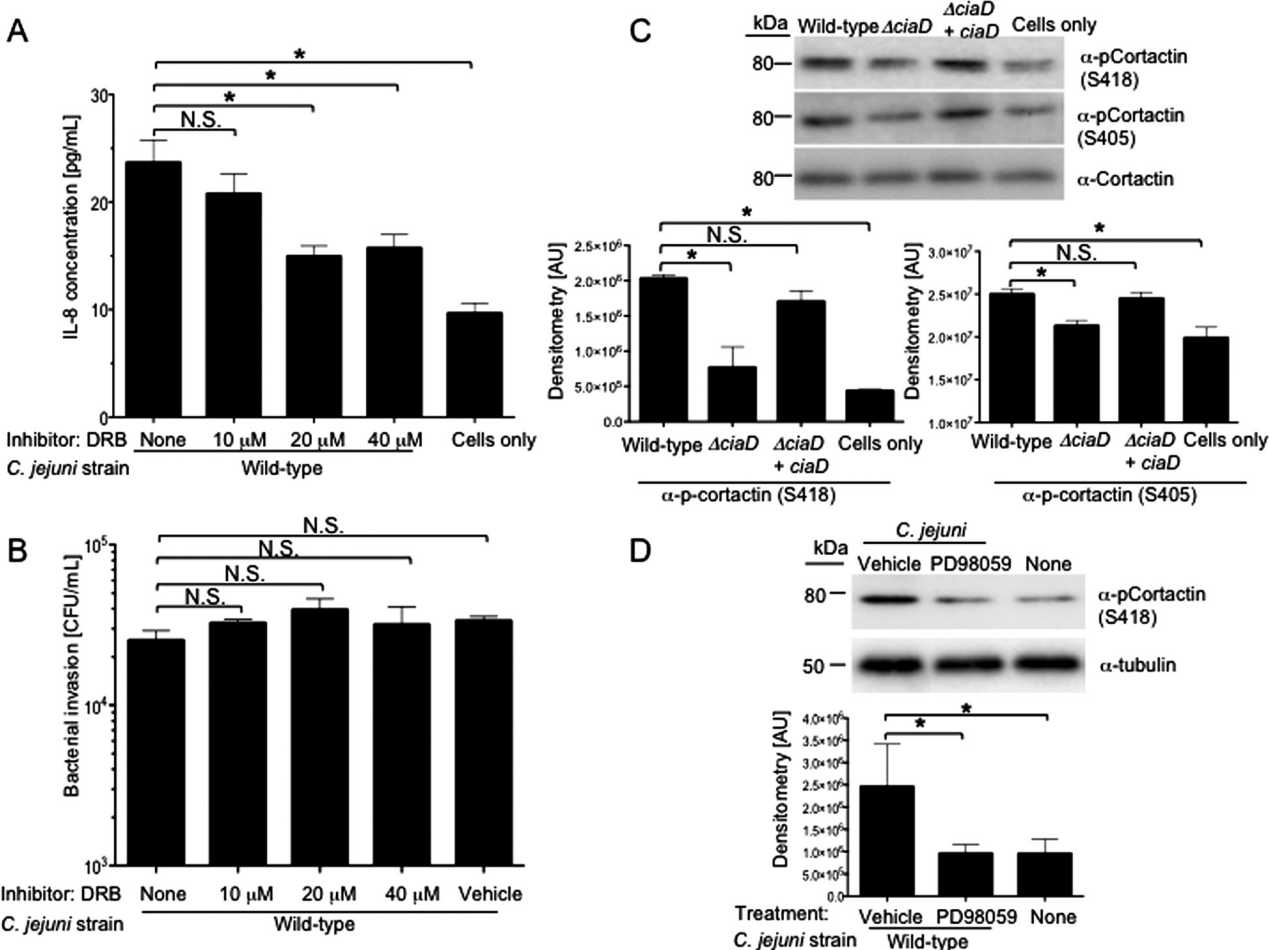
**Erk 1/2 is necessary for cytosolic signaling required for maximal *C. jejuni *invasion of host cell. A**. INT 407 cells were infected with *C. jejuni* incubated for 6 h, and IL-8 quantified using an IL-8 ELISA. The transcription inhibitor 5,6-dichloro-1-beta-D-ribofuranosylbenzimidazole (DRB) was added to INT 407 cells for 30 min prior to infection with a *C. jejuni* wild-type strain. **B**. Transcription is not required for *C. jejuni* invasion. INT 407 cells were infected with *C. jejuni* and invasion was assessed. **C**. CiaD is required for serine phosphorylation of cortactin. INT 407 cells were infected with the various *C. jejuni* strains and cellular lysates were prepared. Blots were probed with phospho-specific antibodies to cortactin. The blot was stripped and re-probed with an α-cortactin antibody. Densitometry of p-cortactin is shown as the ratio of p-cortactin to total cortactin (t-cortactin) for each sample. **D**. Erk 1/2 is required for serine phosphorylation of cortactin. INT 407 cells were pre-treated with PD98059, an inhibitor of Erk 1/2 activation, and infected with a *C. jejuni* wild-type strain. Blots were probed with a phospho-specific antibody to cortactin. The blot was stripped and re-probed with an α-tubulin antibody. Molecular masses, in kilodaltons (kDa), are indicated on the left. The asterisks indicate a significant difference (*P* < 0.01) compared to the value obtained for the *C. jejuni* wild-type strain, as judged by one-way ANOVA followed by post-hoc Dunnett’s analysis. N.S. indicates no significant difference.

Experiments were performed to determine if Erk 1/2 participates in transcriptional regulation of genes and/or activation of cytosolic signaling proteins necessary for actin cytoskeleton rearrangement, leading to *C. jejuni* host cell invasion and membrane ruffling. We first assessed the role of Erk 1/2 mediated transcriptional regulation in *C. jejuni* invasion of host cells. To prevent Erk 1/2 mediated transcriptional activation in response to infection with *C. jejuni,* host INT 407 cells were pre-treated with 5,6-dichloro-1-beta-D-ribofuranosylbenzimidazole (DRB). DRB inhibits Cdk-activating kinase (a TFIIH-associated kinase), thereby preventing transcription by RNA polymerase II [29]. To determine the concentration of DRB necessary to inhibit transcription, cells were pre-treated with different concentrations of DRB and the secretion of interleukin-8 (IL-8) from host cells was determined. The host cell chemokine IL-8 is transcribed, translated, and secreted from host cells in response to numerous bacterial pathogens, including *C. jejuni*[30-35]. Pretreatment of INT 407 cells with DRB resulted in a reduction in the amount of IL-8 in supernatants from *C. jejuni* infected cells (Figure 3A), suggesting that DRB effectively blocks the transcription of IL-8. However, pretreatment of INT 407 cells with DRB resulted in normal *C. jejuni* invasion of host cells (Figure 3B). These results suggest that Erk 1/2 mediated transcriptional regulation is not involved in host cell actin cytoskeleton rearrangement necessary for *C. jejuni* host cell invasion.

Given that Erk 1/2 mediated transcriptional regulation is not required for cytoskeleton rearrangement, we performed experiments to determine if cytosolic signaling mediated by Erk 1/2 was altered or impaired. We chose to investigate the Erk 1/2 mediated phosphorylation of the cytosolic actin binding protein cortactin, a known target of Erk 1/2 and a component of the actin polymerization and nucleation complex [36]. In contrast to infection of INT 407 cells with a *C. jejuni* wild-type strain, the *C. jejuni ciaD* mutant was deficient in maximal phosphorylation of cortactin at the Erk 1/2 phosphorylation sites S405 and S418, as judged by immunoblot analysis with the S418 and S405 phospho-specific antibodies to cortactin (Figure 3C). INT 407 cells infected with the *C. jejuni ciaD* complemented isolate restored the phosphorylation of cortactin to levels indistinguishable from infection with a *C. jejuni* wild-type strain (Figure 3C). This finding indicates that CiaD mediated activation of Erk 1/2 leads to the phosphorylation of cortactin on serine residues. Consistent with the fact that CiaD mediates Erk 1/2 activation and Erk 1/2 mediates the phosphorylation of cortactin on S405 and S418, we found that pretreatment of INT 407 cells with the MEK 1/2 inhibitor PD98059 reduced phosphorylation of cortactin on S418 in response to *C. jejuni* infection, similar to the level observed in uninfected cells (Figure 3D). The inhibition of cortactin serine phosphorylation by treatment of cells with PD98059 is in agreement with published data [37]. However, this is the first report showing that cortactin becomes activated in response to *C. jejuni* infection. Given that CiaD is required for maximal cortactin activation, we assessed the role of cortactin phosphorylation in *C. jejuni* invasion of host cells.

### Cortactin serine phosphorylation is required for maximal invasion

**Figure 4:**
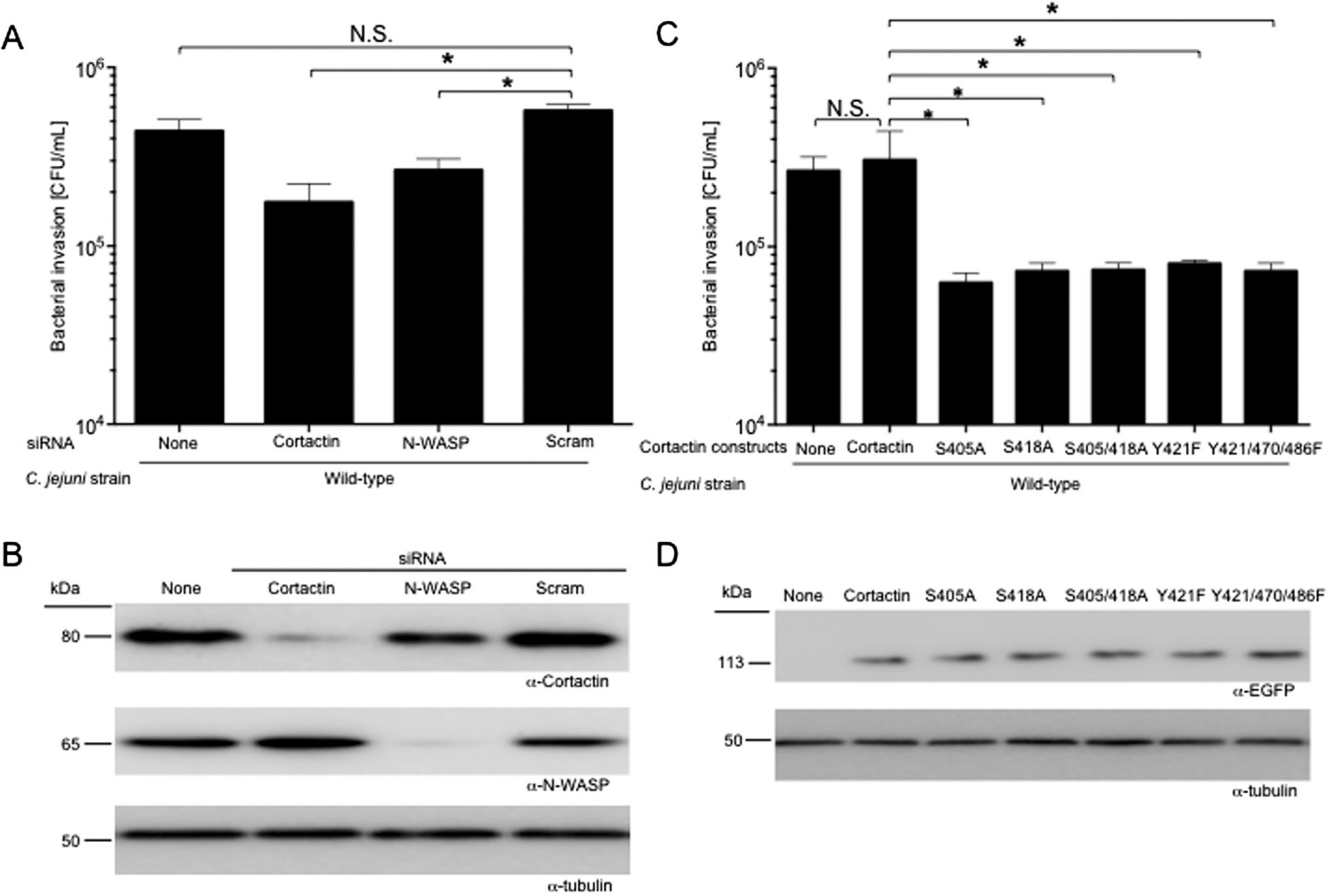
**Knockdown of endogenous cortactin and N-WASP prevent *C. jejuni*-invasion of INT 407 cells. A**. Internalization of *C. jejuni* in INT 407 cells transfected with siRNA to cortactin, siRNA to N-WASP or a scrambled (Scram) siRNA. Results are shown as the mean number of internalized bacteria ± SEM. **B**. Whole cell lysates of untreated, siRNA to cortactin, siRNA to N-WASP, and scramble siRNA-transfected cells were probed with α-cortactin and α-N-WASP antibodies. The blot was re-probed with an α-tubulin antibody to confirm equal loading. **C**. Internalization of *C. jejuni* in INT 407 cells transfected with phosphorylation null constructs of cortactin. Bacterial invasion was assessed using the gentamicin protection assay. Results are displayed as mean number of internalized bacteria ± SEM. **D**. Whole cell lysates of untreated and cortactin phosphorylation null transfected cells were collected and probed with an α-EGFP antibody. The blot was re-probed with an α-tubulin antibody to determine loading. The asterisks indicate a significant difference (*P* < 0.01) compared to the value obtained for the *C. jejuni* wild-type strain, as judged by one-way ANOVA followed by post-hoc Dunnett’s analysis. N.S. indicates no significant difference.

To determine if cortactin is required for *C. jejuni* invasion of host cells, we used small interfering RNA (siRNA) to knockdown cortactin and siRNA to knockdown the downstream complex protein N-WASP. N-WASP is a known component of the actin nucleation and polymerization complex and is necessary for the complete activation of Arp 2/3. More specifically, serine phosphorylation of cortactin leads to the recruitment of N-WASP, activation of Arp 2/3, and actin remodeling [36]. INT 407 cells were transfected with siRNA to cortactin or siRNA to N-WASP, and *C. jejuni* invasion of host cells was evaluated using the gentamicin protection assay*.* The knockdown of cortactin resulted in a significant reduction in the number of *C. jejuni* internalized by host cells (Figure 4A). The knockdown of N-WASP also significantly reduced the amount of internalized *C. jejuni* (Figure 4A). Effective knockdown of cortactin (60.6% ± 3.3% knockdown) and N-WASP (67.8% ± 1.1% knockdown) was demonstrated by immunoblot analysis (Figure 4B). These data support the proposal that cortactin and N-WASP are necessary for maximal *C. jejuni* invasion of host cells.

To evaluate the specific contribution of Erk 1/2 phosphorylation of cortactin at S405 and S418 in *C. jejuni* host cell invasion, phosphorylation null constructs of cortactin were utilized and the gentamicin protection assay was performed. INT 407 cells were transfected with cortactin-EGFP phosphorylation null constructs with the following mutations: S405A, S418A, and S405/418A (double serine mutant). The contributions of c-Src (tyrosine) phosphorylation of cortactin were also evaluated, as c-Src phosphorylation of cortactin is known to be important for the invasion of other pathogens [2,3,5]. INT 407 cells were transfected with cortactin-EGFP Y421F and Y421/470/486 F (triple tyrosine mutant) mutant constructs to evaluate the role of c-Src phosphorylation of cortactin. We found that both cortactin serine and tyrosine phosphorylation are required for maximal invasion of host cells by *C. jejuni,* as judged by the gentamicin protection assay (Figure 4C)*.* Equal expression of the cortactin-EGFP phosphorylation null constructs was confirmed via immunoblot analysis (Figure 4D). In support of the finding that tyrosine phosphorylation of cortactin is required for *C. jejuni* invasion, inhibition of the upstream kinase c-Src with the inhibitor PP2 prevented *C. jejuni* internalization (Additional file 1: Figure S1). This is the first report to our knowledge demonstrating that serine phosphorylation of cortactin by Erk 1/2 and tyrosine phosphorylation of cortactin by c-Src are required for *C. jejuni* invasion of host cells. Based on these results, we hypothesized that cortactin and the serine phosphorylation of cortactin are necessary for *C. jejuni-*induced membrane ruffling.

### Cortactin serine phosphorylation is required for host cell membrane ruffling

To evaluate the role of cortactin activation by CiaD in *C. jejuni*-mediated host cell membrane ruffling, we utilized EGFP-tagged cortactin to visualize membrane ruffling*.* INT 407 cells, which had been transfected with a cortactin-EGFP construct, were infected with the *C. jejuni* wild-type strain, *ciaD* mutant, and the *ciaD* complemented isolate and the cells examined by confocal microscopy. Uninfected cells transfected with cortactin-EGFP exhibited diffuse cortactin localization with no distinct membrane ruffling of cell borders (Additional file 2: Figure S2 A-D). However, infection with the *C. jejuni* wild-type stain resulted in membrane ruffling (Additional file 2: Figure S2 E-H). In contrast, the *C. jejuni ciaD* mutant was deficient in membrane ruffling and exhibited diffuse cortactin localization similar to that of uninfected cells (Additional file 2: Figure S2 I-L). Host cell membrane ruffling was restored when cells were infected with the *C. jejuni ciaD* complemented isolate (Additional file 2: Figure S2 M-P). While this experiment indicated that CiaD is necessary for *C. jejuni*-induced membrane ruffling, it was not clear if cortactin is required for membrane ruffling.

**Figure 5:**
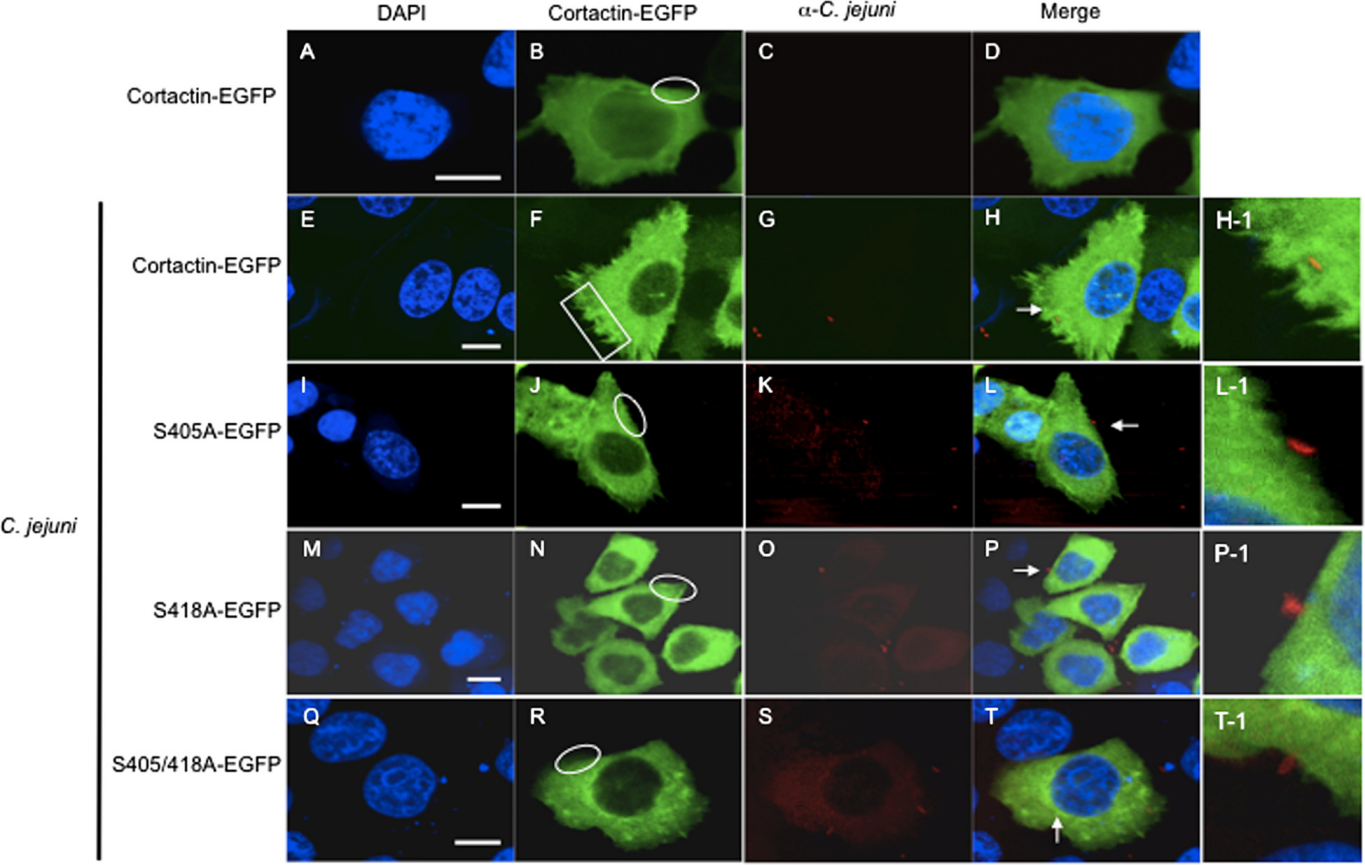
**Phosphorylation null constructs of cortactin prevent *C. jejuni *induced membrane ruffling. A-T**. *C. jejuni* induced membrane ruffling is impaired in INT 407 cells transfected with cortactin S405A, S418A or S405/418A phosphorylation null constructs. Representative confocal microscopy images of INT 407 cells uninfected (Panel **A-D**) and cells infected with the *C. jejuni* wild-type strain with various treatment conditions. The panels represent: Wild-type cortactin-EGFP (Panel **E-H**), cortactin S405A phosphorylation null construct (Panel **I-L**), cortactin S418A phosphorylation null construct (Panel **M-P**), and cortactin S405/418A phosphorylation null construct (Panel **Q-T**). Images from left to right show, DAPI staining of cell nuclei (Panels **A**, **E**, **I**, **M**, and **Q**), EGFP-cortactin (Panel **B**, **F**, **J**, **N**, and **R**), *C. jejuni* staining with a polyclonal rabbit α-*Campylobacter* antibody and a secondary Texas-Red antibody (Panels **C**, **G**, **K**, **O**, and **S**), and merge of all panels (Panels **D**, **H**, **L**, **P**, and **T**). INT 407 cells that display extensive membrane ruffling (Panel **H**), and cells that display no host cell membrane ruffling (Panels **D**, **L**, **P**, and **T**). *C. jejuni* in contact with the host cell is shown in (Panels **H-1**, **L-1**, **P-1**, and **T-1**). Images were obtained with a 63× objective and have a 10 μM scale bar (Panels **A-T**). Arrows indicate *C. jejuni* interaction with host cells*.* The areas within the box highlights regions of membrane ruffling and the areas within the circles indicate regions of no membrane ruffling.

Experiments were performed to determine if serine phosphorylation of cortactin is necessary for *C. jejuni-*induced membrane ruffling. INT 407 cells, which had been transfected with cortactin-EGFP S405A, S418A, and S405/418A phosphorylation null constructs, were infected with a *C. jejuni* wild-type strain and evaluated by confocal microscopy. Uninfected cells transfected with cortactin-EGFP exhibited diffuse cortactin localization with no distinct membrane ruffling of the cell borders (Figure 5A-D). Infection of cortactin-EGFP transfected cells with the *C. jejuni* wild-type strain resulted in distinct membrane ruffling (Figure 5E-H). *C. jejuni* infection of INT 407 cells transfected with EGFP-cortactin S405A (Figure 5I-L), S418A (Figure 5M-P), and S405/418A (Figure 5Q-T) phosphorylation null constructs resulted in a diffuse localization of cortactin and no observable membrane ruffling in response to *C. jejuni* was observed. These results indicate that cortactin is required for *C. jejuni*-induced membrane ruffling.

**Figure 6:**
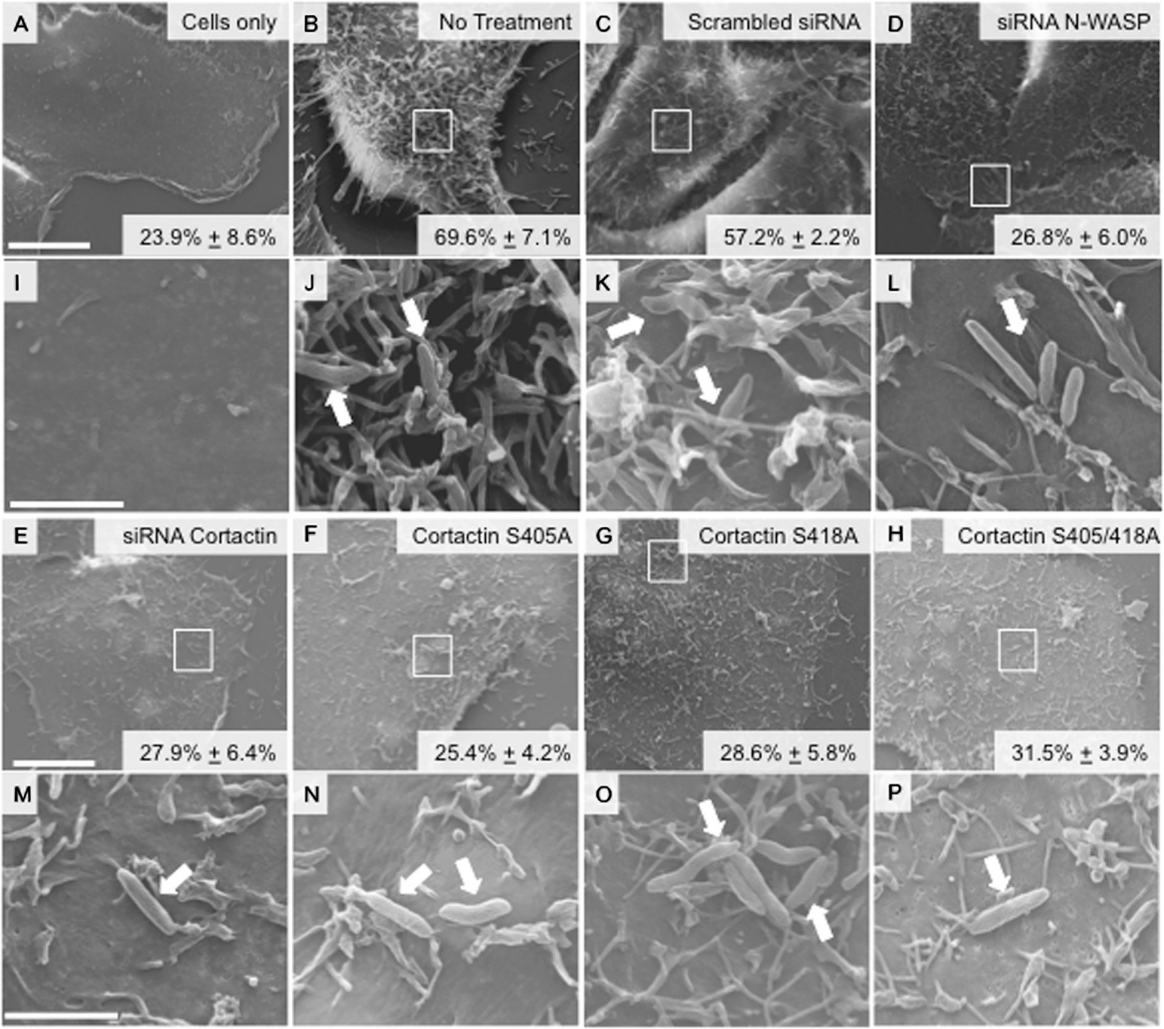
**Knockdown of endogenous cortactin and N-WASP prevent *C. jejuni *induced membrane ruffling. A-P**. *C. jejuni* induced membrane ruffling in INT 407 cells transfected with scrambled (Scram) siRNA, siRNA to N-WASP, siRNA to cortactin, and cortactin S405A, S418A and S405/418A phosphorylation null constructs. Representative scanning electron microscopy images of INT 407 cells uninfected (Panel **A**) and cells infected with *C. jejuni* wild-type strain with various treatment conditions; No treatment (Panel **B**), Scrambled siRNA control (Panel **C**), siRNA to N-WASP (Panel **D**), siRNA to cortactin (Panel **E**), cortactin S405A (Panel **F**), cortactin S418A (Panel **G**), and cortactin S405/4118A (Panel **H**). Arrows in the higher magnification images show *C. jejuni* in contact with host cells (Panels **I-P**). Boxes indicate the area of the INT 407 cell that is shown in the 50,000× panel. INT 407 cells that display extensive membrane ruffling (Panel **J** and **K**), and INT 407 cells that display no host cell membrane ruffling (Panels **L-P**). Images are shown at a magnification of 7,000× with a 10 μM scale bar (Panels **A-H**), and 50,000× with a 2 μM scale bar (Panels **I-P**). Also indicated within each panel is the percent of host cell that display membrane ruffling*.*

Scanning electron microscopy was performed to determine the extent of *C. jejuni-*induced membrane ruffling in cells transfected with siRNA to cortactin and siRNA to N-WASP. More specifically, INT 407 cells were transfected with a scrambled siRNA, siRNA to cortactin, or siRNA to N-WASP, and infected with a *C. jejuni* wild-type strain. INT 407 cells were also transfected with cortactin-EGFP S405A, S418A, and S405/418A phosphorylation null constructs. Representative images of *C. jejuni* interaction with host cells are shown in Figure 6A-P. We observed that 23.9% ± 8.6% of untreated and uninfected INT 407 cells had membrane ruffling (Figure 6A). Membrane ruffling was observed in 69.6% ± 7.1% of the cells infected with a *C. jejuni* wild-type strain (Figure 6B). Treatment of INT 407 cells with a non-coding scrambled siRNA prior to infection with *C. jejuni* resulted in levels of membrane ruffling similar to untreated cells infected with *C. jejuni* (Figure 6C). Treatment of host cells with siRNA to N-WASP and siRNA to cortactin reduced membrane ruffling to 26.8% ± 6.0% and 27.9% ± 6.4%, respectively (Figure 6D and E). We also observed a significant decrease in host cell membrane ruffling when cells were transfected with phosphorylation null constructs of cortactin (Figure 6F-H). Specifically, the cortactin-EGFP S405A, S418A, and S405/418A phosphorylation null constructs reduced membrane ruffling to 25.4% ± 4.2%, 28.6% ± 5.8%, and 31.5% ± 3.9%, respectively. Noteworthy is that treatment of INT 407 cells with siRNA or phosphorylation null constructs prevented membrane ruffling from occurring even in the presence of direct bacterial contact (compare panels J and K to panels L-P). These data indicate that cortactin and N-WASP are required for maximal membrane ruffling induced by *C. jejuni*.

### Cortactin, Erk 1/2, and N-WASP complex formation is CiaD dependent

**Figure 7:**
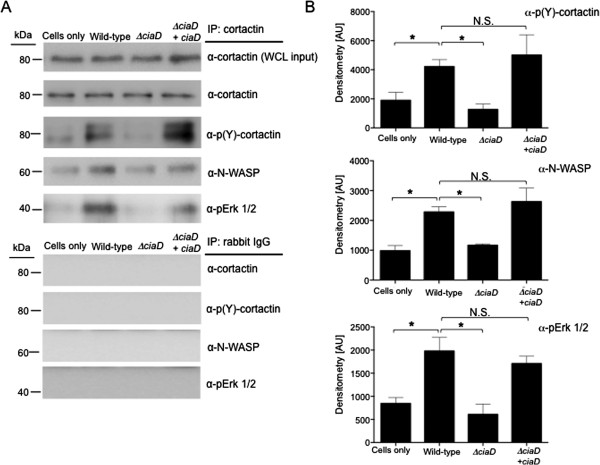
**CiaD is required for Erk 1/2-cortactin association.** INT 407 cells were infected with a *C. jejuni* wild-type strain, *ciaD* mutant, *ciaD* complemented isolate, or uninfected (control) for 45 min. **A**. The cell lysates were subjected to immunoprecipitation experiments with an antibody against cortactin, separated by SDS-PAGE and blotted for cortactin, p-cortactin, N-WASP, and pErk 1/2. Whole cell lysates (WCL) were also probed with an α-cortactin antibody to confirm similar inputs. Also shown are the blots of the IgG isotype control IP probed with cortactin, p-cortactin, N-WASP and pErk 1/2 antibodies. **B**. Band intensity of p-cortactin, N-WASP, and pErk 1/2 were normalized to total cortactin from three independent experiments. The asterisks indicate a significant difference (*P* < 0.01) compared to the value obtained for the *C. jejuni* wild-type strain, as judged by one-way ANOVA followed by post-hoc Dunnett’s analysis. N.S. indicates no significant difference.

Immunoblot and immunoprecipitation (IP) experiments were performed to determine if cortactin and Erk 1/2 are associated in response to *C. jejuni* infection. INT 407 cells were infected with *C. jejuni* for 45 minutes and cortactin was precipitated using an α-cortactin antibody. Infection of INT 407 cells with a *C. jejuni* wild-type strain resulted in a significant increase (above uninfected cells) in the amount of phosphorylated (activated) cortactin (Figure 7). In contrast, the amount of activated cortactin significantly decreased in INT 407 cells infected with the *C. jejuni ciaD* mutant. Specifically, infection with the *C. jejuni* wild-type strain and the *ciaD* complemented isolate resulted in a significant increase in the level of activated cortactin, but the *C. jejuni ciaD* mutant was indistinguishable from uninfected cells (Figure 7). The association of Erk 1/2 and cortactin in cells infected with the *C. jejuni* wild-type strain, *ciaD* mutant, and *ciaD* complemented isolate was also determined by immunoprecipitation*.* We found that CiaD is required for the maximal association of cortactin with phosphorylated Erk 1/2 (Figure 7). The association of N-WASP with cortactin was also determined to occur in a CiaD dependent manner (Figure 7). This finding suggests that N-WASP is associated with the serine phosphorylated form of cortactin. Others have shown that serine phosphorylation of cortactin is necessary for N-WASP association [36,38-40]. The IP experiments performed also revealed that cortactin associates with phospho-Erk 1/2 and N-WASP upon *C. jejuni* infection, and that N-WASP and phospho-Erk 1/2 association with cortactin is dependent on CiaD (Figure 7). These data show that *C. jejuni* induces the formation of the actin nucleation and polymerization complex Erk 1/2-cortactin-N-WASP, and that this association is, in part, dependent of the *C. jejuni* effector protein CiaD. These data also show that the recruitment of N-WASP to cortactin requires Erk 1/2 serine phosphorylation of cortactin.

## Discussion

**Figure 8:**
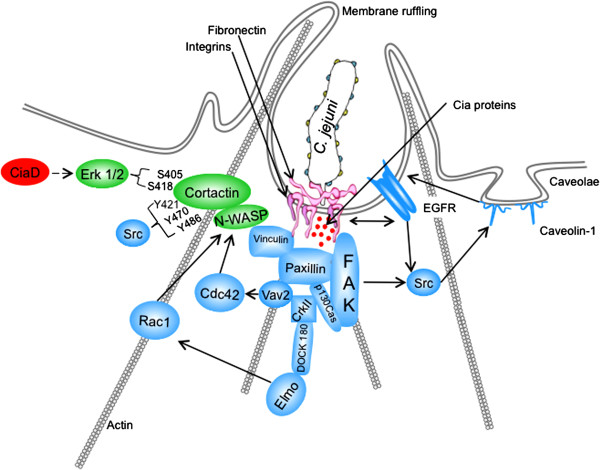
**Model of *C. jejuni *internalization.***C. jejuni* invasion of host cells. Step 1: *C. jejuni* binds to fibronectin (Fn) via the two *C. jejuni* Fn binding proteins CadF (blue dots) and FlpA (yellow dots) [43,44] causing activation of the α_5_β_1_ integrin receptors and the epidermal growth factor receptor (EGFR) [27,45]. Step 2: Activation of the α_5_β_1_ integrin leads to the recruitment and partial activation of FAK and paxillin [27,46]. Step 3: The delivery of the *Campylobacter* invasion antigens (*e.g.*, CiaD shown in red) to the host cell [16,19,27,47] leads to the maximal activation of key components of the focal complex (*i.e.*, FAK, paxillin, vinculin, p130Cas, Src, and the CrkII/DOCK-180/ELMO complex) [27,28,45, Konkel *et. al,* Invasion of epithelial cells by *Campylobacter jejuni* is independent of caveolin-1, In Submission]. Step 4: Focal complex activation, in conjunction with CiaD, leads to the phosphorylation of Erk 1/2. Caveolin-1, Vav2, Rac1, and Cdc42 are also activated following focal complex activation [27,28,45]. Step 5: Activation of Erk 1/2 and Src leads to the phosphorylation of cortactin, which allows for the Rho GTPases Rac1 and Cdc42 to activate N-WASP associated with phosphorylated cortactin, promoting actin cytoskeletal reorganization. Highlighted in this model is the role of CiaD in *C. jejuni* internalization. Specifically, CiaD is necessary for the maximal activation of the Erk 1/2 and cortactin signaling pathways. Components of the focal complex and focal complex associated proteins are shown in blue. The newly identified components of the *C. jejuni* invasion complex are shown in green.

This study was performed to further elucidate the mechanism of *C. jejuni* invasion of host cells. More specifically, we investigated the role of Erk 1/2 and cortactin in *C. jejuni* invasion of host cells. Erk 1/2 is a serine/threonine kinase that is part of the Ras-Raf-MEK-ERK signal transduction cascade. Erk 1/2 is activated by dual phosphorylation at Y204/187 and T202/185 catalyzed by MEK 1/2 [41,42]. Erk 1/2 catalyzes the phosphorylation of hundreds of cytoplasmic and nuclear proteins and participates in numerous cellular processes including cell adhesion, cell cycle progression, cell migration, cell survival, differentiation, metabolism, proliferation, and transcription [42]. Cortactin is a filamentous actin binding protein that is a crucial link between the organization of structural proteins, such as actin, and cellular signal transduction pathways. Cortactin stimulates actin polymerization via interaction with N-WASP through its SH3 domain, and binding of Arp 2/3 through its N-terminal domain [36]. Cortactin is regulated by phosphorylation of Y421, Y470, and Y486 by c-Src and other tyrosine kinases [36]. Likewise, Erk 1/2 phosphorylates S405 and S418 of cortactin [36,38]. There is also evidence that PAK phosphorylates cortactin, however the implications of PAK serine phosphorylation are poorly defined [1,36]. Work by Martinez-Quiles *et al.* (2004) revealed that phosphorylation of cortactin by Erk 1/2 acts as a positive regulatory event and Src phosphorylation acts as a negative regulatory event in actin cytoskeletal rearrangement by activation/deactivation of N-WASP and Arp2/3 [40]. Additionally, Kelley *et al.*[39] demonstrated that concurrent phosphorylation of cortactin by Erk 1/2 and tyrosine kinases allow cells to regulate actin dynamics through N-WASP. Taken together, it is clear that the activation and deactivation of cortactin by phosphorylation is a dynamic process. In the present study, we showed that phosphorylation of cortactin on S405, S418, Y421, Y470, and Y486 are required for maximal invasion of host cells by *C. jejuni*. Specifically, we show that CiaD is required for maximal activation of Erk 1/2 (Figure 1). Activation of Erk 1/2 leads to the phosphorylation of S405 and S418 on cortactin (Figure 3). Also, the association of cortactin with Erk 1/2 is dependent on CiaD (Figure 7). Furthermore, we found that serine phosphorylation of cortactin is required for maximal *C. jejuni* induced host cell membrane ruffling. These findings provide the basis for a detailed model of *C. jejuni* invasion of host cells (Figure 8).

Previous work has shown that Dock180 and its binding partner ELMO (Engulfment and Cell Motility) form a bipartite guanine nucleotide exchange factor (GEF), resulting in the activation of Rac1 and membrane ruffling [27]. *C. jejuni* invasion of cells is also accompanied by the activation of Cdc42 [28,45]. Interestingly, several effector proteins from *Salmonella enterica*, including SopE, SopE2 and SopB, modulate the activity of Cdc42 and Rac1 to manipulate actin cytoskeleton rearrangements [48,49]. Noteworthy is that the IcsA effector protein from *Shigella flexneri* promotes filopodia formation by binding and activating N-WASP in a Cdc42-like fashion [50]. To determine the role of CiaD in *C. jejuni* invasion of host cells, we first evaluated the Rho GTPases Rac1 and Cdc42 as a loss in the activation of either protein could explain the invasion deficiency of the *ciaD* mutant. INT 407 cells infected with the *C. jejuni ciaD* mutant exhibited levels of Rho GTPase activation similar to that of cells infected with the *C. jejuni* wild-type strain. This is in stark contrast to cells infected with a *C. jejuni ciaC* mutant that display a significant reduction in Rac1 activation [27]. The reduction in Rac1 activity with the *C. jejuni ciaC* mutant is in agreement with the fact that there are fewer sites of co-localized Rac1 in INT 407 cells infected with the *C. jejuni ciaC* mutant versus a *C. jejuni* wild-type strain [27]. Our data supports the proposal that CiaD is manipulating cellular signaling cascades and altering actin nucleation at a site downstream from Rac1 and Cdc42. Moreover, our results indicate that CiaC and CiaD manipulate at least two distinct host cell targets that are necessary for *C. jejuni* invasion of host cells.

Based on the observation that Erk 1/2 is critical for *C. jejuni* invasion of host cells, we performed experiments to determine if: 1) Erk 1/2 is transcriptionally regulating cellular components involved in cell invasion; and/or 2) Erk 1/2 is necessary for the activation of cytosolic cellular signaling cascades involved in cytoskeleton rearrangement. We found that the transcription of the gene that encodes for IL-8 is not required for invasion, but that Erk 1/2 is required for the serine phosphorylation of cortactin. As previously stated, cortactin is an actin-binding protein that recruits N-WASP and activates Arp 2/3, leading to actin remodeling [36]. Interestingly, Erk 1/2 activation stimulates bacterial capture of *Shigella* by filopodia [51], while the OspF effector protein from *Shigella* harbors phosphatase activity to inactivate mitogen-activated protein kinases (MAPKs), including Erk 1/2, c-Jun N-terminal kinase, and p38, post-invasion [52]. Collectively, these data highlight the fact that Erk 1/2 is a key component of the *C. jejuni* invasion complex and that bacterial pathogens can manipulate membrane extensions (*e.g.*, lamellipodia and filopodia) by targeting Erk 1/2.

Cortactin is likely involved in the uptake of pathogenic bacteria into host cells, as it acts in concert with N-WASP to activate the Arp2/3 complex. It is plausible for a pathogen to activate cortactin directly or to activate cortactin indirectly via Erk 1/2 or Src. For example, the IpaC effector protein from *Shigella* mediates Src-dependent phosphorylation of cortactin, thereby promoting actin polymerization [53]. We found that serine phosphorylation of cortactin by *C. jejuni* is dependent upon Erk 1/2, as the level of phospho-cortactin in *C. jejuni* infected cells treated with the PD98059 inhibitor was indistinguishable from uninfected cells.

Here we demonstrate that the formation of the Erk 1/2-cortactin-N-WASP complex is dependent on the *C. jejuni* effector protein CiaD. To our knowledge this is the first report of the involvement of cortactin and N-WASP in host cell invasion by *C. jejuni*. In addition, this is the first report showing that Erk 1/2 mediated serine phosphorylation of cortactin is required for *C. jejuni* invasion of host cells. Future studies will focus on the identification of the direct target of CiaD and the kinetics and regulation of cortactin in bacterial invasion. This work provides new insight into *C. jejuni* pathogenesis and the complex signaling events exploited by bacterial pathogens during the process of invasion. Equally important, it contributes to the understanding of the phosphorylation of cortactin in bacterial infection.

## Conclusion

In this study, we present a model for *C. jejuni* invasion of host cells (Figure 8). We show that *C. jejuni* activates Erk 1/2. Specifically, we found that Erk 1/2 activation is dependent on the *C. jejuni* effector protein CiaD. Similarly, we found that Erk 1/2 activation is necessary for bacterial invasion because it is necessary for the phosphorylation of serine residues in cortactin. Moreover, inhibition of serine phosphorylation results in decreased bacterial invasion and host cell membrane ruffling. The requirement of serine phosphorylation of cortactin by Erk 1/2 in *C. jejuni* host cell invasion represents a mechanistic basis for how Erk 1/2 inhibition leads to impaired *C. jejuni* invasion.

## Methods

### Bacterial strains, tissues culture types

The *Campylobacter jejuni* F38011 clinical isolate was used in this study. All *C. jejuni* isolates were cultured on Muller-Hinton agar plates containing bovine citrated blood (MHB) with the appropriate antibiotic at the following final concentrations: chloramphenicol 8 microgram/mL and tetracycline 2 microgram/mL. Cultures were grown at 37°C in a microaerobic chamber (5% O_2_, 10% CO_2_, and 85% N_2_). INT 407 cells (ATCC CCL 6) were obtained form the American Type Culture Collection (ATCC). INT 407 cells were cultured in minimal essential medium (MEM) supplemented with 10 mM sodium pyruvate, 20 mM glutamine, and 10% (v/v) fetal bovine serum (FBS).

### Preparation of INT 407 whole cell lysates

Whole cell lysates (WCL) of INT 407 cells were prepared by the addition of lysis buffer, as described previously [27]. The lysates were collected and analyzed by SDS-PAGE coupled with immunoblot analyses. The protein concentration of each sample was determined by the bicinchoninic acid (BCA, Pierce, Rockford, IL) protein assay and normalized prior to SDS-PAGE.

### Immunoblot analysis, cellular inhibitors, antibodies, and densitometry analysis

Immunoblots were performed as described previously [27]. INT 407 cell lysates were collected and analyzed by SDS-polyacrylamide gel electrophoresis. The proteins were transferred to a polyvinylidene fluoride (PVDF) membrane, and probed with the indicated antibodies. Primary antibodies reactive against N-WASP (Cell Signaling Technology, Cat # 4848), phospho-Erk 1/2 (Cell Signaling Technology, Cat # 4377), total-Erk 1/2 (Santa Cruz, Dallas, TX, Cat # sc-94) cortactin (Cell Signaling Technology, Cat # 3503), phospho-cortactin (S418) (Protea, Morgantown, WV, Cat # AB-110), phospho-cortactin (S405) (Protea Biosciences, Inc., Morgantown, WV, Cat # AB-100), tyrosine-phospho-cortactin (Millipore, Billerica, MA, Cat # 05–180) and tubulin (Sigma, Cat # T6199) were used at a 1:1000 dilution and incubated overnight at 4°C. The α-rabbit IgG or the α-mouse IgG (Sigma, St. Louis, MO) secondary antibodies were applied at a 1:2000 dilution for 1 h at room temperature. Band intensity was quantified using a LAS 4000 mini (GE healthcare) and the Multi Gauge V3.0 (Fujifilm, Valhalla, NY) software package. Densitometry analysis is shown as the ratio of phosphoprotein to total protein (*i.e*., p-Erk 1/2 to total Erk 1/2 and p-cortactin to total cortactin) or as the ratio of target protein to cortactin in the IP experiments (*i.e.,* N-WASP to total cortactin and p-Erk 1/2 to total cortactin). Inhibitors or vehicle [dimethyl sulfoxide (DMSO)] were added to INT 407 cells 30 min prior to infection and maintained throughout the assay. The Erk 1/2 inhibitor PD98059 (Selleck, Houston, TX, Cat # S1177) was used at 50 μM. The c-Src inhibitor PP2 (Sigma, Cat # P0042) was used at 5, 10, 20, and 40 μg/mL. The transcription inhibitor 5,6-dichloro-1-beta-D-ribofuranosylbenzimidazole (DRB) (Sigma, Cat # D1916) was used at 10, 20, and 40 μM. Cell death was quantified with trypan blue staining. No significant death of INT 407 cells was observed with any of the treatments (not shown).

### Binding and internalization assays

Binding and internalization assays were performed as described elsewhere [27]. INT 407 cells were seeded at a density of 1.5 × 10^5^ into 24-well flat bottom tissue culture trays (BD Falcon, Franklin Lakes, NJ, Cat # 353047). Bacteria were suspended in MEM containing 1% FBS and added to cells at a multiplicity of infection (MOI) of 100. Trays were centrifuged at 800 × *g* to promote bacterial cell contact. Cells were lysed with 0.1% Triton-X100 and plated onto MHB agar for bacterial enumeration. *C. jejuni* host cell invasion was assessed by lysing INT 407 cells and enumerating the internalized bacteria following a 3 h incubation with 250 microgram/mL of gentamicin.

### Transfection of phosphorylation null constructs

Human Cortactin-GFP and S405A, S418A, S405/S418A, Y421F, and Y421/470/486F phosphorylation null GFP constructs of cortactin were generously provided by Dr. Scott Weed from West Virginia University [38]. Plasmids were purified using the Qiagen Plasmid Purification Kit (Qiagen, Valencia, CA) according to the manufacturer’s protocols. Purified plasmids were quantified using NanoDrop 2000c (Thermo Scientific, Wilmington, DE) and normalized to 200 ng/μl. Purified plasmids where transfected into INT 407 cells seeded on glass coverslips at 3 × 10^5^. Transfections where performed using the Qiagen Effectene Transfection reagent (Qiagen, Valencia, CA), according to the manufacturer’s specifications.

### Confocal microscopy

INT 407 cells were infected with *C. jejuni* for 45 min at 37°C in a 5% CO_2_ incubator prior to fixation with 3.7% paraformaldehyde for 15 min. *C. jejuni* were stained with a 1° rabbit α-*C. jejuni* antibody (Konkel Laboratory Collection) and a 2° Texas Red dye-conjugated donkey α-rabbit antibody (Jackson ImmunoResearch Labs, West Groves, PA). The coverslips were mounted with VectaShield and 4′,6-diamidino-2-phenylindole (DAPI, Vector Laboratories, Burlingame, CA) added to stain DNA. Images were obtained using a Zeiss confocal microscope using a 63X, 1.4 NA water immersion objective lens.

### Scanning electron microscopy

Scanning electron microscopy was performed as described elsewhere [27]. INT 407 cells were transfected with the phosphorylation null or siRNA constructs. *C. jejuni* was added to cells for 15 minutes. Quantification of membrane ruffling was done by two independent observers and tabulated. Cells were counted and cells positive for membrane ruffling were scored.

### Treatment of cells with small interfering RNA (siRNA)

INT 407 cells were transfected with siRNA using lipofectamine RNAiMAX (Invitrogen, Grand Island, NY) according to the manufacturer’s instructions. Cortactin stealth siRNA (Invitrogen, Grand Island, NY, Cat # S4665), N-WASP stealth siRNA (Invitrogen, Cat # S17132), and scrambled control siRNA (Invitrogen, Cat # 46–2000) were applied to the cells 24 h prior to infection. Knockdown of endogenous proteins were confirmed by immunoblot.

### Assessment of Rho GTPase Rac1 and Cdc42 activation

INT 407 cells were seeded into 6-well tissue culture trays at a density of 2 × 10^5^ cells/well and serum starved for 24 h. *C. jejuni* was resuspended in PBS and added to the cells. The amount of activated Rac1 and Cdc42 in *C. jejuni*-infected and uninfected cells was determined using the G-LISA™ Rac1 and Cdc42 Activation Assays according to the manufacturer’s instructions (Cytoskeleton, Denver, CO).

### Immunoprecipitation

INT 407 cells were seeded at 3 × 10^6^ cells per dish and serum starved in MEM for 3 h prior to the addition of *C. jejuni* or the uninfected (negative control). Forty-five min post-infection, cells were collected in ice-cold lysis buffer as described previously [27]. Immunoprecipitations were performed by incubating cell lysates with an α-cortactin antibody (Cell Signaling Technology, Inc., Danvers, MA) at 4°C overnight and then adding protein A/G beads at 4°C for 1 h with rotation. The bead complexes were washed and dissolved in sample buffer.

### IL-8 quantification

Interleukin-8 levels in cellular supernatants were quantified with a commercial ELISA (OptEIA Set, Becton Dickinson, Cowley, Oxford, UK) using the manufacturer’s protocol. INT 407 cells were pre-treated with DRB as described above and inoculated with *C. jejuni.* Cells were centrifuged for 5 min at 800 × *g* to promote cell contact. The cells were incubated at 37°C for 6 h and the media were collected from each well. Supernatants were used immediately or frozen at −20°C.

### Statistical analysis

All data were evaluated using one-way ANOVA followed by post-hoc Tukey’s or Dunnet’s analysis of the means, using Prism 6 (GraphPad Software, La Jolla, CA). Statistical significance was defined by a maximum value of *P *< 0.05.* All experiments were performed a minimum of three times to ensure reproducibility.

## Abbreviations

ERK: Extracellular regulated kinase; MAPK: Mitogen activated protein kinase; N-WASP: Neuronal Wiskott-Aldrich protein; Cia: *Campylobacter* invasion antigens.

## Competing interests

Authors declare no conflicts of interest.

## Authors’ contributions

DRS planned experiments, performed experiments, and wrote the manuscript. MEK planned experiments, analyzed data, and contributed to preparation of the manuscript. Both authors read and approved the final manuscript.

## Supplementary Material

Additional file 1: Figure S1 Inhibition of c-Src prevents *C. jejuni* invasion of INT 407 cells. Internalization of *C. jejuni* by INT 407 cells treated or untreated (control) with the c-Src inhibitor PP2 for 30 min prior to bacterial invasion. Bars represent the mean number of internalized bacteria ± SEM. The asterisks indicate a significant difference (*P* < 0.01) compared to the value obtained for the *C. jejuni* wild-type strain, as judged by one-way ANOVA followed by post-hoc Dunnett’s analysis. Click here for file

Additional file 2: Figure S2 CiaD is required for membrane ruffling. A-P. CiaD is required for *C. jejuni* induced membrane ruffling in INT 407 cells transfected with cortactin-EGFP. Representative confocal microscopy images of INT 407 cells uninfected (Panel A-D) and infected with a *C. jejuni* wild-type strain (Panel E-H), a *ciaD* mutant (Panel I-L), and a *ciaD* complemented isolate (Panel M-P). Images from left to right show, DAPI staining of cell nuclei (Panels A, E, I, and M), EGFP-cortactin (Panel B, F, J, and N), *C. jejuni* staining with a polyclonal rabbit α-*Campylobacter* antibody and a secondary Texas-Red (Panels C, G, K, and O), and merge of all panels (Panels D, H, L, and P). *C. jejuni* in contact with the host cell is shown in Panels H-1, L-1, and P-1. Images were taken with a 63× objective and have a 10 μM scale bar (Panels A-P). Arrows indicate *C. jejuni* interaction with host cells*.* The areas within the boxes highlight regions of membrane ruffling (Panels F and N) and the areas within the circles indicate regions of no membrane ruffling (Panels B and J). Click here for file
