# Supplementary figures and images for: Serine phosphorylation of cortactin is required for maximal host cell invasion by Campylobacter jejuni
Source: Cell Commun Signal. 2013 Nov 4;11:82. doi: 10.1186/1478-811X-11-82 (PMC3832248; doi:10.1186/1478-811X-11-82)

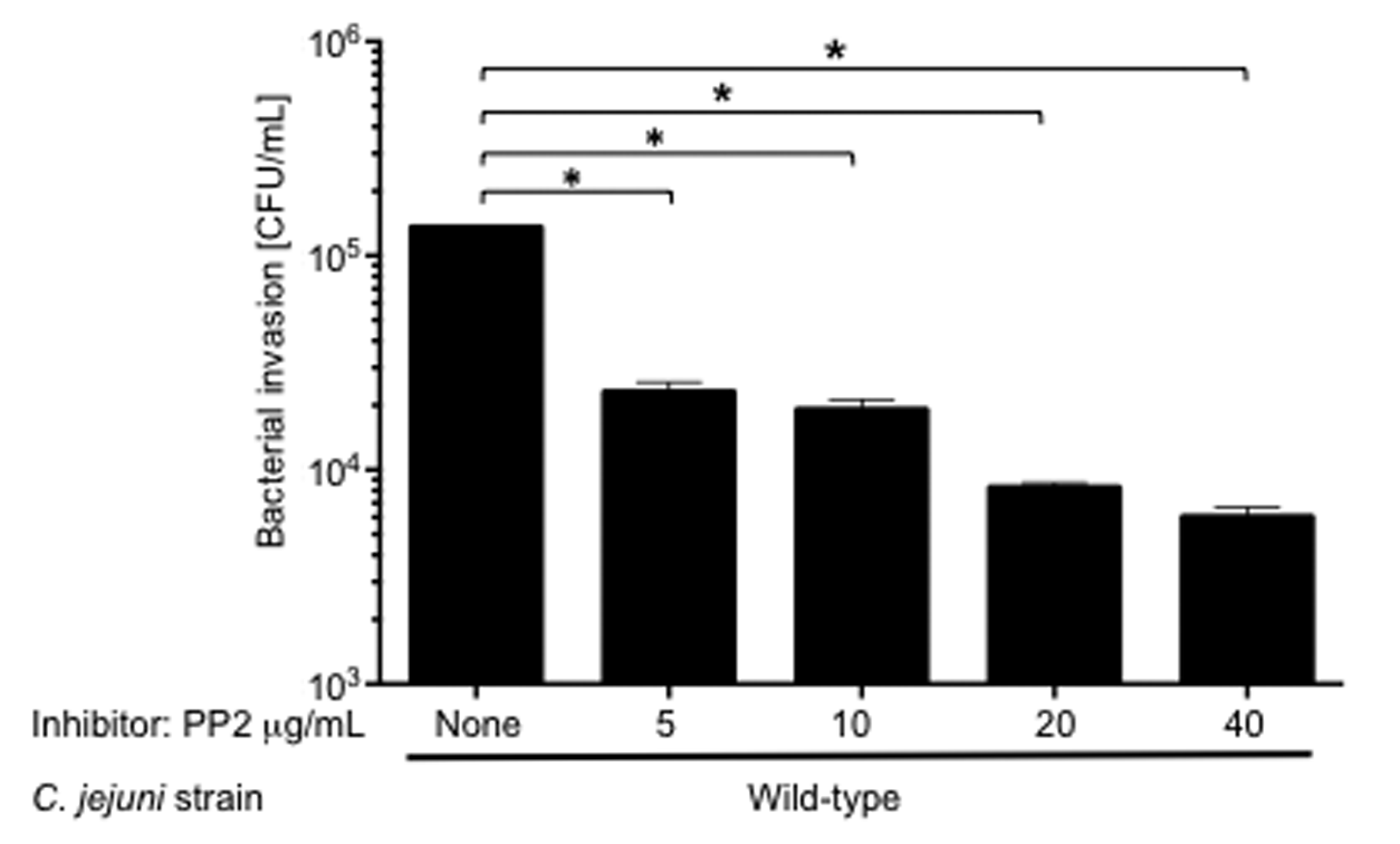

Supplement: Additional file 1: Figure S1 — Inhibition of c-Src prevents C. jejuni invasion of INT 407 cells. Internalization of C. jejuni by INT 407 cells treated or untreated (control) with the c-Src inhibitor PP2 for 30 min prior to bacterial invasion. Bars represent the mean number of internalized bacteria ± SEM. The asterisks indicate a significant difference (P < 0.01) compared to the value obtained for the C. jejuni wild-type strain, as judged by one-way ANOVA followed by post-hoc Dunnett’s analysis. [file 1478-811X-11-82-S1.tiff]

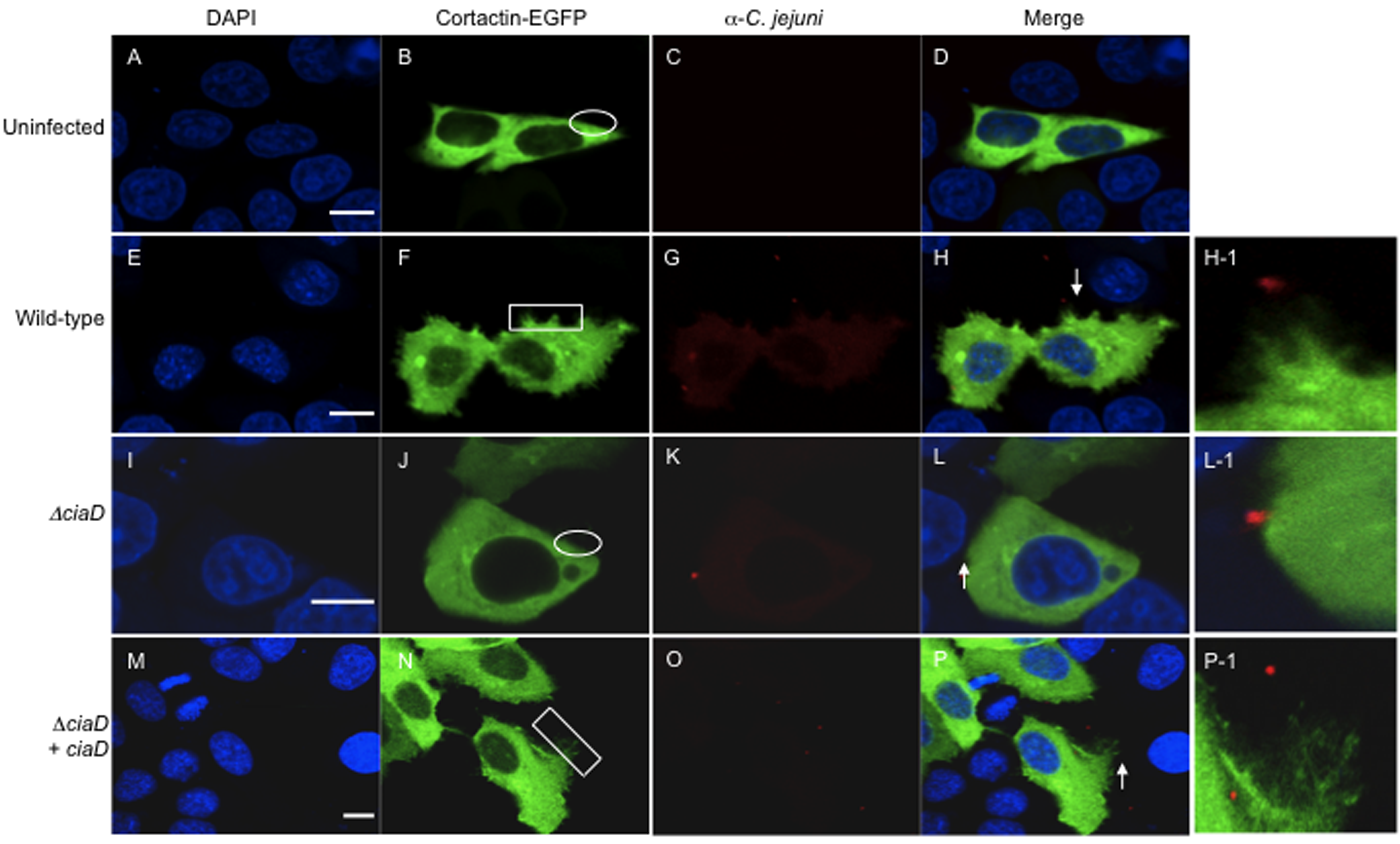

Supplement: Additional file 2: Figure S2 — CiaD is required for membrane ruffling. A-P. CiaD is required for C. jejuni induced membrane ruffling in INT 407 cells transfected with cortactin-EGFP. Representative confocal microscopy images of INT 407 cells uninfected (Panel A-D) and infected with a C. jejuni wild-type strain (Panel E-H), a ciaD mutant (Panel I-L), and a ciaD complemented isolate (Panel M-P). Images from left to right show, DAPI staining of cell nuclei (Panels A, E, I, and M), EGFP-cortactin (Panel B, F, J, and N), C. jejuni staining with a polyclonal rabbit α-Campylobacter antibody and a secondary Texas-Red (Panels C, G, K, and O), and merge of all panels (Panels D, H, L, and P). C. jejuni in contact with the host cell is shown in Panels H-1, L-1, and P-1. Images were taken with a 63× objective and have a 10 μM scale bar (Panels A-P). Arrows indicate C. jejuni interaction with host cells. The areas within the boxes highlight regions of membrane ruffling (Panels F and N) and the areas within the circles indicate regions of no membrane ruffling (Panels B and J). [file 1478-811X-11-82-S2.tiff]
